# 初诊多发性骨髓瘤患者骨髓多克隆浆细胞占比与临床特征的相关性分析

**DOI:** 10.3760/cma.j.cn121090-20231020-00221

**Published:** 2024-05

**Authors:** 筱露 龙, 欣然 王, 宁 安, 松雅 刘, 喆 李, 春晖 李, 伟 穆, 迪 王, 春蕊 李

**Affiliations:** 华中科技大学同济医学院附属同济医院血液内科，武汉 430030 Department of Hematology, Tongji Hospital, Tongji Medical College, Huazhong University of Science and Technology, Wuhan 430030, China

**Keywords:** 多发性骨髓瘤, 浆细胞, 预后, Multiple myeloma, Plasma cells, Prognosis

## Abstract

**目的:**

探究初诊多发性骨髓瘤（NDMM）患者骨髓多克隆浆细胞占比（pPC％）与临床特征的相关性。

**方法:**

回顾性分析2018年1月至2023年1月在华中科技大学同济医学院附属同济医院收治的317例NDMM患者，纳入患者均有明确的pPC％结果。分析pPC％与临床特征的关系。

**结果:**

共纳入317例患者，其中男180例，女137例，中位确诊年龄61（26～91）岁，≥60岁患者占55.8％。NDMM患者骨髓pPC％在各DS分期、ISS分期、R-ISS分期组差异均有统计学意义（*P*值分别为0.002、0.010、0.049），而不同的FISH危险分层患者pPC％差异无统计学意义（*P*＝0.971）。NDMM患者pPC％与患者初诊时HGB呈正相关（*r*＝0.211，*P*<0.01）；与血钙、血肌酐、单克隆免疫球蛋白（M蛋白）定量、β_2_微球蛋白水平呈负相关（*r*分别为−0.141、−0.120、−0.181、−0.207，*P*值为0.012、0.033、0.004、0.002）。与pPC％≥2.5％患者相比，pPC％<2.5％组患者初诊时受累轻链、血钙、血肌酐、M蛋白和β_2_微球蛋白水平明显升高（*P*值均<0.05），HGB降低（*P*<0.001），ISS分期Ⅲ期比例更高（*P*＝0.034）。

**结论:**

NDMM患者pPC％与良好预后的临床特征相关，包括更高的HGB，更低的血钙、血肌酐、M蛋白定量、β_2_微球蛋白，受累轻链，更低的晚期疾病占比（DS分期、ISS分期Ⅲ期），在临床特点上表现为肿瘤负荷较小。

## Abstract (en)

**Objective:**

To explore the correlation of bone marrow polychonal plasma cell proportion（pPC％）and clinical features in newly diagnosed multiple myeloma（NDMM）patients.

**Methods:**

A retrospective analysis of 317 patients with NDMM admitted to Tongji Hospital, Tongji Medical College, Huazhong University of Science and Technology from January 2018 to January 2023 was performed. The results of the pPC％ in all patients were clear. The relationship between the pPC％ and clinical characteristics was analyzed.

**Results:**

A total of 317 patients were included, comprising 180 males and 137 females. The median age at diagnosis was 61（26–91）years, and 55.8％ were 60 years or older. The pPC％ in the bone marrow of patients with NDMM was different in the DS, International Staging System（ISS）, and revised ISS（R-ISS）stages（*P*＝0.002, 0.010, and 0.049, respectively）, whereas no statistical difference in pPC％ was observed among patients with different FISH risk stratigrams（*P*＝0.971）. The correlation coefficient between pPC％ and hemoglobin（HGB）at the first diagnosis in patients was 0.211（*P*<0.01）. The correlation coefficients with serum calcium, serum creatinine, M protein level, and β_2_-microglobulin were −0.141, −0.120, −0.181, and −0.207, respectively, and the results of the significance test were *P*＝0.012, 0.033, 0.004, and 0.002, respectively, indicating a negative correlation. Compared with the patients with a pPC％ of ≥2.5％, the group of patients with a pPC％ of <2.5％ had significantly higher levels of light chain, serum calcium, serum creatinine, M protein, and β_2_-microglobulin at the initial diagnosis（*P*<0.05）; lower HGB level（*P*<0.001）; and a higher proportion of patients in ISS stage Ⅲ（*P*＝0.034）.

**Conclusion:**

In this study, the pPC％ in patients with NDMM was associated with clinical features of good prognosis, including higher HGB, lower serum calcium, serum creatinine, M protein quantity, β_2_-microglobulin, light chain involvement, lower proportion of advanced disease（DS stage and ISS stage Ⅲ）, and clinical features showing lower tumor burden.

多发性骨髓瘤（MM）是一种浆细胞克隆性恶性增生性疾病，特征为异常浆细胞在髓内和髓外的单克隆扩增，同时伴有大量单克隆免疫球蛋白或轻链增多[1]。正常浆细胞是体液免疫的重要组成部分，它们能产生绝大部分的血清抗体，有助于抵御广泛的病原体和毒素[2]。当出现恶性克隆性浆细胞大量增殖时，正常浆细胞会受抑制。

多克隆浆细胞（polyclonal plasma cell，pPC）在MM中的潜在生物学意义尚不清楚，需要进一步研究。与活动期MM患者相比，治疗后疾病控制期长达10年以上的MM患者，其体内pPC水平较高[3]。这些正常pPC可能在维持正常的骨髓微环境中发挥作用，从而阻止恶性转化和单克隆浆细胞的增殖[4]。然而，初诊MM（NDMM）患者的pPC与其临床特征的关系，以及pPC占骨髓浆细胞总数的比例对MM患者的影响目前报道较少，本研究对317例NDMM患者进行了回顾性分析，旨在评估NDMM伴有pPC患者的临床特征。

## 病例与方法

1. 病例资料：本研究共纳入317例2018年1月至2023年1月在华中科技大学同济医学院附属同济医院收治的NDMM患者，纳入患者均具有初诊时流式细胞术（FCM）检测结果，并有明确的多克隆浆细胞占比（pPC％）结果。本研究经我院伦理委员会批准（批件号：TJ-IRB20231294），并获得患者的知情同意。

2. 诊断及分期标准：MM诊断标准参照国际骨髓瘤工作组（IMWG）2016年的指南及《血液病诊断及疗效标准（第4版）》。临床分期参照《中国多发性骨髓瘤诊治指南（2022年修订）》，采用Durie-Salmon（DS）分期及国际骨髓瘤工作组提出的国际分期系统（ISS）标准进行分期，FISH检测细胞遗传学异常分为高危和低危组，定义参照2022年修订的中国多发性骨髓瘤诊疗指南[5]–[9]。

3. FCM：FCM采用多抗体联合检测，应用CD45/CD38、CD45/CD138设门找出浆细胞。正常浆细胞免疫表型为CD45^+^/CD38^+^/CD138^+^/CD19^+^/CD56^−^，多克隆表达cLambda、cKappa。骨髓瘤细胞免疫表型常为CD45^dim/−^/CD38^+^/CD138^+^/CD19^−^/CD56^+^，限制性表达cKappa或cLambda。

4. 随访：随访截止日期为2023年7月，以查阅患者住院病历及电话联系的方式进行随访。总生存（OS）期定义为自患者接受治疗到因任何原因死亡的时间，无进展生存（PFS）期定义为自患者接受治疗到疾病进展或因任何原因死亡的时间。

5. 统计学处理：计数资料采用百分比表示，符合正态分布的计量资料采用*x*±*s*表示，不符合正态分布的计量资料采用*M*（*Q*_1_，*Q*_3_）表示。应用SPSS 25.0和Graphpad Prism 9.0软件进行统计学分析。患者临床特征的比较采用Mann-Whitney *U*检验、*χ*^2^检验或Fisher精确概率法，相关性采用Spearman相关分析，组间比较使用Bonferroni校正得到矫正*P*值。以上均为双侧检验，以*P*<0.05为差异有统计学意义。

## 结果

1. 基本临床特征：本研究共纳入317例患者，其中男180例，女137例，患者中位确诊年龄为61（26～91）岁，60岁及以者的占55.8％。中位pPC％为0.30％，其中完全不伴有pPC（pPC％＝0）和伴有pPC（pPC％>0）的患者分别为101例和216例。明确DS分期的患者共292例，其中Ⅰ期31例（10.6％），Ⅱ期25例（8.6％），Ⅲ期236例（80.8％）；明确ISS分期的患者共244例，其中Ⅰ期44例（18％），Ⅱ期86例（35.2％），Ⅲ期114例（46.7％）。243例患者进行了FISH检测并进行危险分层，其中标危组157例（64.6％），高危组86例（35.4％）（表1）。

**表1:** 317例初诊多发性骨髓瘤患者的基本临床特征及其pPC％均值

临床特征	例数（%）	患者pPC%均值（%）	*P*值
年龄			0.618
<60岁	140（44.2）	4.84	
≥60岁	177（55.8）	5.54	
性别			0.316
男	180（56.8）	5.18	
女	137（43.2）	5.12	
分型^a^			0.077
IgG型	153（48.4）	11.48	
IgA型	85（26.9）	2.95	
轻链型	56（17.7）	2.28	
IgD/E型	13（4.1）	1.10	
不分泌型	7（2.2）	9.37	
双克隆型	2（0.6）	6.00	
DS分期^b^			0.002
Ⅰ期	31（10.6）	11.28	
Ⅱ期	25（8.6）	3.37	
Ⅲ期	236（80.8）	4.16	
ISS分期^c^			0.010
Ⅰ期	44（18.0）	9.88	
Ⅱ期	86（35.2）	6.15	
Ⅲ期	114（46.7）	3.34	
R-ISS分期^d^			0.049
Ⅰ期	27（11.9）	8.96	
Ⅱ期	140（61.9）	5.82	
Ⅲ期	59（26.1）	3.37	
FISH危险分层^e^			0.971
标危	157（64.6）	4.51	
高危*	86（35.4）	3.63	

**注** ^a^例数为316例，^b^例数为292例，^c^例数为244例，^d^例数为226例，^e^例数为243例；*细胞遗传学高危指间期荧光原位杂交检出del（17p）、t（4;14）、t（14;16）。pPC％：多克隆浆细胞占比；ISS：国际分期系统；R-ISS：修订版国际分期系统

2. NDMM患者骨髓pPC％与MM分型、分期、危险分层的关系：在有血清M蛋白类型数据的316例患者中IgG型pPC％均值最高（11.48％），大于IgA型pPC％均值（2.95％）和轻链型pPC％均值（2.28％），其余分型例数较少。患者骨髓pPC％在各DS分期（*P*＝0.002）、ISS分期（*P*＝0.010）、R-ISS分期（*P*＝0.049）组差异均具有统计学意义，而不同FISH危险分层患者pPC％差异无统计学意义（*P*＝0.971）（表1）。同时，单克隆浆细胞占比在不同DS分期（*P*＝0.003）、ISS分期（*P*＝0.016）患者中差异均有统计学意义，而在不同R-ISS分期（*P*=0.058）和FISH危险分层（*P*＝0.928）组单克隆浆细胞占比差异无统计学意义。

进一步对不同分期下pPC％进行组间两两统计比较（表2）：Ⅰ期患者pPC％均明显高于Ⅲ期患者（DS分期*P*＝0.002，ISS分期*P*＝0.028，R-ISS分期*P*＝0.042）。而在单克隆浆细胞占比中，DS分期（*P*＝0.002）和ISS分期（*P*＝0.037）Ⅰ期低于Ⅲ期，而在R-ISS分期中差异无统计学意义。

**表2:** 初诊多发性骨髓瘤患者的多克隆浆细胞和单克隆浆细胞占比在不同的分期组间比较

浆细胞占比	组间比较	*P*值		
		DS分期	ISS分期	R-ISS分期
多克隆浆细胞占比	Ⅰ期与Ⅱ期	0.351	1.000	0.204
	Ⅰ期与Ⅲ期	0.002	0.028	0.042
	Ⅱ期与Ⅲ期	0.815	0.056	0.687
单克隆浆细胞占比	Ⅰ期与Ⅱ期	0.350	1.000	0.197
	Ⅰ期与Ⅲ期	0.002	0.037	0.051
	Ⅱ期与Ⅲ期	0.885	0.083	0.836

**注** ISS：国际分期系统；R-ISS：修订版国际分期系统

此外，对不同分期下pPC与单克隆浆细胞的比值进行了Mann-Whitney *U*检验，组间比较使用Bonferroni校正得到矫正*P*值，pPC与单克隆浆细胞的比值（pPC/mPC）在DS分期、ISS分期、R-ISS分期组间比较，三组差异均有统计学意义（DS分期*P*＝0.002，ISS分期*P*＝0.015，R-ISS分期*P*＝0.048），并且在组间两两比较中均发现DS分期（*P*＝0.002）和R-ISS分期（*P*＝0.048）中Ⅰ期明显低于Ⅲ期。

3. NDMM患者骨髓pPC％与临床特征的相关性分析：对pPC％与临床检验指标进行相关性分析发现，NDMM患者pPC％与患者初诊时血红蛋白浓度之间呈正相关（*r*＝0.211，*P*<0.01）；pPC％与相应的血钙、血肌酐、M蛋白定量、β_2_微球蛋白水平呈负相关，*r*分别为−0.141、−0.120、−0.181、−0.207，*P*值分别为0.012、0.033、0.004、0.002；pPC％与受累轻链、白蛋白、LDH水平无相关性。

4. 比较不同水平的pPC％对NDMM患者临床特征的影响：以pPC％＝0为分界值，pPC％＝0和pPC％>0两组患者临床特征差异均无统计学意义（*P*>0.05）；参考此前大部分研究pPC％的分界值，以pPC％＝5％为分界值，与pPC≥5％患者相比，pPC％<5％组患者初诊时受累轻链、血钙、血肌酐、M蛋白和β_2_微球蛋白水平明显更高（*P*值均<0.05），血红蛋白水平降低（*P*<0.001），DS分期和ISS分期均为Ⅲ期比例更高（DS分期*P*<0.001，ISS分期*P*＝0.003）（表3）；以DS分期Ⅰ+Ⅱ期与Ⅲ期（*P*＝0.002）为预测结局，以pPC％为变量做ROC曲线，取约登指数（Youden index）最大的点作为cut-off值，AUC＝0.632，2.5％作为cut-off值，灵敏度为80.1％，特异性为44.6％，准确率为73.3％（图1A～B）。

**图1:**
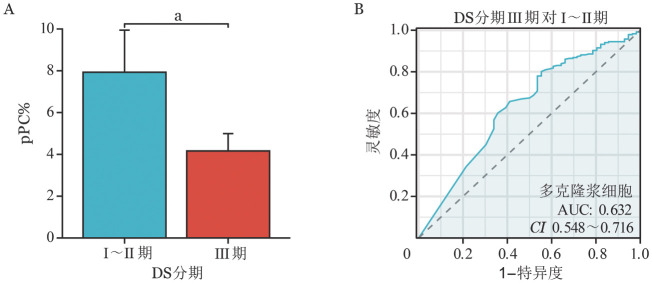
初诊多发性骨髓瘤患者的DS分期Ⅰ～Ⅱ期与Ⅲ期组多克隆浆细胞占比（pPC％）比较（A）及ROC曲线图（B） **注** ^a^*P*<0.01

以pPC％＝2.5％为分界值，与pPC％≥2.5％患者相比，pPC％<2.5％组患者初诊时受累轻链、血钙、血肌酐、M蛋白和β_2_微球蛋白水平明显更高（*P*值均<0.05），血红蛋白水平降低（*P*<0.001），ISS分期Ⅲ期比例更高（*P*＝0.034）（表3）。

**表3:** 不同pPC％分界（0％、2.5％、5％）下初诊多发性骨髓瘤患者骨髓pPC％与临床特征的关系

临床特征	pPC%=0（101例）	pPC%>0（216例）	*P*值	pPC%<2.5%（235例）	pPC%≥2.5%（82例）	*P*值	pPC%<5%（256例）	pPC%≥5%（61例）	*P*值
DS期[例（%）]			0.165			<0.001			<0.001
Ⅰ期	6（2.1）	25（8.6）		14（4.8）	17（5.8）		17（5.8）	14（4.8）	
Ⅱ期	6（2.1）	19（6.5）		17（5.8）	8（2.7）		20（6.8）	5（1.7）	
Ⅲ期	81（27.7）	155（53.1）		189（64.7）	47（16.1）		203（69.5）	33（11.3）	
ISS期[例（%）]			0.117			0.034			0.003
Ⅰ期	10（4.1）	34（13.9）		29（11.9）	15（6.1）		31（12.7）	13（5.3）	
Ⅱ期	20（8.2）	66（27.0）		58（23.8）	28（11.5）		63（25.8）	23（9.4）	
Ⅲ期	40（16.4）	74（30.3）		93（38.1）	21（8.6）		102（41.8）	12（4.9）	
受累轻链[*M*（*Q_1_*,*Q_3_*）]	254.80 (119.28, 464.50)	220.25 (64.28, 424.25)	0.351	260.00 (96.45, 465.00)	190.90 (61.00, 303.00)	0.045	260.00 (96.45, 464.00)	186.30 (57.70, 284.62)	0.029
HGB（*x*±*s*）	88.153±26.356	93.403±27.587	0.112	87.565±26.118	103.660±27.129	<0.001	87.656±25.828	108.820±26.706	<0.001
血钙[*M*（*Q_1_*,*Q_3_*）]	2.44 (2.31, 2.80)	2.45 (2.29, 2.59)	0.287	2.45 (2.31, 2.70)	2.39 (2.24, 2.55)	0.014	2.45 (2.31, 2.68)	2.33 (2.22, 2.55)	0.003
肌酐[*M*（*Q_1_*,*Q_3_*）]	98.50 (72.75, 188.50)	86.00 (69.00, 132.25)	0.163	93.00 (72.00, 167.75)	82.50 (68.00, 112.00)	0.031	93.00 (71.50, 169.00)	82.00 (68.00, 102.00)	0.008
M蛋白[*M*（*Q_1_*,*Q_3_*）]	31.28 (13.22, 65.50)	27.76 (9.80, 56.31)	0.263	38.71 (14.58, 62.96)	16.51 (5.81, 47.51)	0.002	34.15 (14.26, 61.14)	16.10 (5.09, 49.18)	0.010
β_2_微球蛋白[*M*（*Q_1_*,*Q_3_*）]	5.94 (3.69, 12.06)	4.83 (3.24, 9.41)	0.125	5.61 (3.55, 13.61)	4.07 (2.91, 6.01)	0.001	5.61 (3.64, 13.81)	3.51 (2.78, 4.86)	<0.001

**注** pPC％：多克隆浆细胞占比；ISS：国际分期系统

5. 预后分析：本研究中位随访时间26.9个月，其中pPC％≥2.5％组患者中位OS期仍未达到，4年OS率为79.35％，中位PFS期为42.2个月，4年PFS率为38.86％；pPC％<5％组患者中位OS期未达到，4年OS率为67.52％，中位PFS期为41.0个月，4年PFS率为25.58％。单因素生存分析显示，两组患者OS（*P*＝0.813）及PFS（*P*＝0.901）的差异均无统计学意义。

## 讨论

Pérez-Persona等[10]证明pPC与恶性单克隆浆细胞之间存在持续和渐进的竞争，在同一时间只有一个群体能在环境中占主导，正如从意义不明的单克隆丙种球蛋白血症（MGUS）和冒烟型骨髓瘤（SMM）发展到症状性MM中观察到的，单克隆浆细胞逐渐取代pPC。先前研究表明pPC％<5％的存在与MGUS和SMM进展为有症状的MM的风险相关[11]。此外，在新诊断和复发的MM患者的骨髓浆细胞（BMPC）中，pPC％>5％均发现与更好的预后相关[12]–[13]。但并没有研究对NDMM患者的BMPC中的pPC进行量化分析。本研究在量化分析的同时尝试引入新的pPC％分界值来探索pPC占比多少对临床特征的影响。

本研究发现NDMM患者pPC％在不同年龄、性别、M蛋白分型组差异均无统计学意义，而在不同DS分期、ISS分期、R-ISS分期组差异均有统计学意义。DS分期主要反映肿瘤负荷与临床进程，而ISS和R-ISS分期主要用于预后判断，即初诊时pPC％越低，出现晚期疾病（DS分期、ISS分期、R-ISS分期Ⅲ期）的比例越高，而该变化趋势侧面印证了其他文献报道的结果：从MGUS或SMM疾病初期进展到症状性MM的过程中，由于骨髓浆细胞生态位有限，单克隆浆细胞与多克隆浆细胞渐进性竞争并取代后者[14]。而高危细胞遗传学对pPC％<5％的MM患者预后的影响在既往报道中存在争议，尽管本研究发现FISH危险分层中标危和高危患者pPC％差异无统计学意义，但单克隆恶性细胞遗传性特征是否影响pPC的存在情况，还需要进一步的研究和实验来判断。

本研究对pPC％与实验室检验指标进行了量化分析，发现NDMM患者pPC％与患者初诊时血红蛋白水平呈正相关，与代表肿瘤负荷的临床指标血钙、血肌酐、M蛋白定量、β_2_微球蛋白呈负相关，而与受累轻链、白蛋白、LDH水平无关。

本研究在尝试找出1个pPC％比例来区分pPC数目对MM患者的影响时，首先根据pPC％＝0％分界，结果显示在临床相关指标和临床分期上，两组之间差异没有统计学意义。因此，仅考虑是否存在pPC缺乏临床指导意义。过去的研究通常将pPC％是否达到5％作为分界值，然而，本研究发现pPC％≥5％的患者占比较少（19.2％），而在pPC％<5％的患者中，临床特征仍存在较大的异质性，因此5％是否是最佳的截断值还有待讨论。为了寻找更合适的分界值，本研究进行了诊断性ROC曲线分析，并得到了新的分界值为2.5％。通过比较在2.5％和5％两种分界下的MM患者临床特征，发现两种分界值分组得到的结论是一致的，即pPC％较高的NDMM患者表现出较高的血红蛋白、更低的血钙、血肌酐、M蛋白定量、β_2_微球蛋白以及受累轻链，并且DS分期、ISS分期Ⅲ期患者比例更低。在新的分界值下，pPC％≥2.5％的患者占到25.8％，与5％分界得到了同样的结论，这可能扩大了预测的范围。

既往研究显示，在新诊断和复发的MM患者的BMPC中，pPC>5％均发现与更好的预后相关[12]–[13]。在本研究中，由于回顾性研究的特点，随访过程中存在较多的失访和数据删失，pPC≥2.5％和pPC<2.5％两组的中位OS期均未达到，中位PFS期分别为42.2个月和41.0个月，单因素生存分析提示两组患者预后差异无统计学意义。然而，在深入分析数据时，我们注意到pPC％≥2.5％组患者4年OS率（79.35％对67.52％）和PFS率（38.86％对25.58％）均比pPC％<2.5％组患者高。此外，此前大部分研究通常将pPC％是否达到5％作为分界值，我们也对pPC≥5％和pPC<5％两组进行了预后分析，发现两种分界值分组得到的结论是一致的。我们认为这可能揭示了pPC％对远期预后的影响，pPC％较高的患者可能与更好的预后相关。

总之，本研究观察到NDMM患者pPC％与良好预后的临床特征相关，包括更高的血红蛋白水平，更低的血钙、血肌酐、M蛋白定量、β_2_微球蛋白，受累轻链，更低的晚期疾病占比（DS分期、ISS分期Ⅲ期），在临床特点上表现为肿瘤负荷较小。然而，作为一项单中心的回顾性研究，本研究的研究人群相对单一，数据可能存在偏倚和混杂因素。还需要进行更多大样本量、多中心的研究，形成更加系统的认知。
